# Navigating heme pathways: the breach of heme oxygenase and hemin in breast cancer

**DOI:** 10.1007/s11010-024-05119-5

**Published:** 2024-09-17

**Authors:** Valeria Consoli, Valeria Sorrenti, Maria Gulisano, Mariarita Spampinato, Luca Vanella

**Affiliations:** 1 Department of Drug and Health Sciences, University of Catania, 95125 Catania, Italy; 2 CERNUT - Research Centre on Nutraceuticals and Health Products, University of Catania, 95125 Catania, Italy

**Keywords:** Breast cancer, Heme oxygenase, Hemin, Ferroptosis, Iron, Cancer therapeutics

## Abstract

Breast cancer remains a significant global health challenge, with diverse subtypes and complex molecular mechanisms underlying its development and progression. This review comprehensively examines recent advances in breast cancer research, with a focus on classification, molecular pathways, and the role of heme oxygenases (HO), heme metabolism implications, and therapeutic innovations. The classification of breast cancer subtypes based on molecular profiling has significantly improved diagnosis and treatment strategies, allowing for tailored approaches to patient care. Molecular studies have elucidated key signaling pathways and biomarkers implicated in breast cancer pathogenesis, shedding light on potential targets for therapeutic intervention. Notably, emerging evidence suggests a critical role for heme oxygenases, particularly HO-1, in breast cancer progression and therapeutic resistance, highlighting the importance of understanding heme metabolism in cancer biology. Furthermore, this review highlights recent advances in breast cancer therapy, including targeted therapies, immunotherapy, and novel drug delivery systems. Understanding the complex interplay between breast cancer subtypes, molecular pathways, and innovative therapeutic approaches is essential for improving patient outcomes and developing more effective treatment strategies in the fight against breast cancer.

## Heme and heme-oxygenase implications in cancer hallmarks

Inorganic iron is known to play key roles in different mechanisms linked to cancer metabolic adaptation and tumor microenvironment reprogramming [1, 2]. Furthermore, iron-containing porphyrin heme, which binds organic iron, can represent a candidate molecule under tumor cells control which can interact with the microenvironment, modulate the energy metabolism and promote proliferation. Heme is an iron protoporphyrin complex fundamental for cell survival due to its involvement in several essential biological processes. However, heme has the potential for representing a toxic and carcinogenic molecule [3]. Heme biological functions go beyond those related to the iron atom presence, among these oxygen transport and storage, drug and steroid metabolism, transcriptional and translational regulation are just same examples [4, 5]. There is a substantial amount of evidence which indicate heme as trigger for carcinogenesis through cytotoxicity induction, reactive oxygen species (ROS) production, immune cell function modulation, together with dysbiosis and inflammation onset [6–8].

Heme oxygenase (HO) serves as the key enzyme responsible for breaking down endogenous heme. It catalyzes the rate-limiting step in this reaction, yielding ferrous ions, carbon monoxide (CO), and biliverdin (BV). BV is subsequently reduced by biliverdin reductase, leading to the formation of bilirubin (BR). HO is also recognized as the primary source of endogenous CO production and represents the exclusive and essential mechanism within cells for heme catabolism. Heme oxygenase can be found in three active isoforms, HO-1, HO-2, and HO-3, respectively. HO-1 is also known as Heat shock protein 32 (Hsp32) and represents the inducible isoform whose expression can be upregulated by several stimuli, including oxidative stress, UV irradiation, infections, heavy metals, cytokines. Besides, HO-2 and HO-3 are considered the constitutive isoforms expressed at basal levels in most of human tissues [9–11]. HOs are categorized as housekeeping genes and play a crucial role in regulating the intracellular heme pool. HO is mainly considered a cytoprotective system, however, when heme levels rise above the detoxification capacity of ferritin and HO-1, it can result in the buildup of free Fe^2+^ and the creation of superoxides, both of which are crucial factors in initiating carcinogenesis [3, 12]. Overall, literature indicates HO system as a double-edged sword which can exert both beneficial and detrimental roles within cellular framework.

Tumorigenesis is caused by different alterations in cellular processes and results in cancerous cells acquiring specific common features which have been described/listed by Hanahan and Weinberg as “The Hallmarks of Cancer”. The proposed hallmarks include sustained proliferative signaling, activation of invasion and metastasis spread, growth suppression systems escape, replicative immortality, induction of angiogenesis, cell death resistance, energetic reprogramming and immune destruction avoidance, together with specific enabling characteristics as genome instability, genome mutation, and tumor-promoting inflammation [13].

Redox homeostasis plays a fundamental role in several biological events, regulating signaling pathways related to cell proliferation, apoptosis, DNA damage, cellular differentiation, and potential chemoresistance [14–16]. Thus, its balance needs to be finely maintained by different detoxifying systems and antioxidant enzymes, including heme and HO, respectively.

Studies have shown a positive correlation between iron and heme levels *in vivo* hypothesizing that iron excess in tumors sustains itself the synthesis of heme, which in turn can affect p53 stability and consequently p53 pro-survival function. Thus, the hypothesis that cancer cells alter not only iron metabolism but overall heme metabolism exerting a regulatory role of p53 expression in order to activate proliferation and cell survival signaling during tumorigenesis, has been formulated [3, 17]. In this context, enhanced heme synthesis in cancer mediates TCA cycle cataplerosis, primarily for the consumption of succinyl-CoA rather than for heme production. Indeed, it was observed that increased heme biosynthesis plays a crucial role in avoiding accumulation of toxic TCA cycle intermediates in hereditary leiomyomatosis and renal-cell cancer [18]. In response, HO-1 levels suffer a significant increase which can sustain cell proliferation mechanisms and escape from cell death. For instance, data reported by Nam et al. demonstrated a direct role of p53 in promoting cell survival through direct transcriptional activation of HO-1 in a specific context [19].

HO-1 overexpression has been observed across various cancer types [20–22] and in rapidly proliferating cells such as epithelial cells in injured skin or psoriatic lesions [23]. Intriguingly, HO-1 has been shown to also influence cell cycle, although its effects are cell-type specific and can be opposite in different tissues [24, 25], indeed HO-1 leverage on cell proliferation can be variable in different types of tumor cells depending on basal expression levels reflecting its superseding role in redox balance and cellular homeostasis. A direct stimulatory effect on proliferation has been demonstrated in different models, for instance downregulation of HO-1 by means of siRNA was associated with a significant inhibition of pancreatic tumor cells growth [26], utilization of HO-1 enzymatic inhibitors showed a strong reduction of prostate, lung and glioblastoma cells proliferation [27] and in murine and human melanoma cell lines HO-1 overexpression resulted in significantly augmented proliferation [28]. Although the mechanism underlying HO-1 ability to modulate cell division programs still needs further elucidation, preliminary studies from Was et al. suggest a potential association of HO-1 overexpression with downregulation of the cell cycle negative regulators p21 and B-cell translocation gene-2 (BTG2) [24, 28]. Conversely, HO-1 induction has been observed to also exert antiproliferative effects on different tumors [29–32] highlighting its potential role in recently described programmed cell death ferroptosis, which has been proposed as novel alternative approach for overcoming apoptosis-inducing drugs resistance in many types of cancer, including BC [33]. Preliminary studies focusing on HO-1 induction reported a significant reduction in colon cancer cell viability following administration of well-known inducer cobalt protoporphyrin (CoPP) through the activation of apoptotic program. The work by Wu et al. reported the occurrence of Casp-3 and PARP-dependent activation of apoptosis following induction of HO-1, demonstrating a correlation between HO-1 activity and colon cancer proliferation through a suggested increase of ER stress especially in those cell lines less differentiated as COLO205, HCT-15 and LOVO [34]. On the other hand, HO-1 is considered to promote immune tolerance and suppression, acting as a mediator in the crosstalk between innate and adaptive immune response. Indeed, HO-1 induction has been proved to protect both cells and tissues from immunological destruction through the promotion of CD4+CD25+ regulatory T cells generation in murine models, showing a prominent immunomodulatory activity [35].

As part of carcinogenesis process, HO-1 upregulation can alter TME leading to reduced cancer cell immune recognition through different mechanisms. Firstly, cancerous cells can upregulate HO-1 in order to elude immune surveillance by consequently altering the expression of membrane receptors to which immune cells adhere or releasing immune-suppressive cytokines. Secondly, immune cells themselves demonstrated to be able to overexpress HO-1 gaining a more tolerant phenotype [36, 37].

Aberrant protein synthesis can be considered a characteristic feature of cancer [38]. Numerous studies have revealed that the tumor phenotype is influenced not only by overall alterations in protein synthesis but also by specific changes in translational efficiency [39]. Furthermore, tumor cells possess the capability to reprogramme the proteome to sustain their transformed phenotype and enhance survival in response to fluctuating nutrient availability and environmental stress [40, 41]. When facing metabolic stress, cells decrease overall protein synthesis to alleviate ER stress [42] promoting the function of activating transcription factor 4 (ATF4), which represent an ER stress key regulator [43]. Among the numerous targets transcriptionally activated by ATF4, HO-1, encoded by the HMOX1 gene, stands out as one of the most extensively studied stress-induced antioxidant enzymes [44]. High HO-1 levels mitigate ER stress and alleviate oxidative stress to facilitate cell resistance to radiation or chemotherapy. Several studies have validated the efficacy of arginine deprivation therapy in breast and prostate cancers, demonstrating its effectiveness through glycolysis reduction and a shift in metabolic pathways. In this context, HO-1 upregulation contributes to improved recovery from metabolic stress induced by arginine deprivation. Therefore, suppressing HO-1 translation through mechanisms triggered by arginine shortage has been suggested as a therapeutic advantage against cancer [45, 46].

Cells undergoing senescence show peculiar features as irreversible growth arrest, increased dimensions and granularity, polyploidization, DNA damage response (DDR) pathway initiation and senescence-associated secretory phenotype (SASP) [47]. Senescence serves as dual modulator in tumor development processes. On one hand, it acts as a powerful tumor-suppressive mechanism. When cells experience oncogenic stress or accumulate extensive DNA damage, they enter a state of permanent growth arrest called senescence. This prevents the proliferation of potentially malignant cells. Thus, many anticancer therapies aim to induce senescence in cancer cells to inhibit their growth. Stress-induced premature senescence (SIPS), intended as an acute type of cellular response independent from telomere erosion, has been proposed as a drug resistance mechanisms [48, 49]. SIPS can be triggered by oxidative stress and DNA damaging agents as conventional chemotherapeutics, in this particular case it can be referred as therapy-induced senescence (TIS)[50]. Thus, drugs targeting the DNA damage response, can push cancer cells into senescence.

On the other hand, while SASP can reinforce growth arrest and attract immune cells to clear senescent cells, it can also create a pro-inflammatory environment that promotes tumor progression and metastasis. Additionally, some cancer cells can bypass the senescence blockade, particularly if they acquire additional mutations. This can lead to treatment resistance and cancer recurrence [51, 52].

In this regard, Borkowska et al. have reported a prominent role of hemin and HO-1 in colorectal cancer cells evading senescence. Relevant findings of their work highlight the ability of hemin, administered at high doses, to induce progression through the G0/G1 phase, and upregulation of proliferation and EMT markers followed by an increase in HO-1 levels. However, conflicting data on HO-1 role in senescent cells have been reported, underlining a specific cell-type/TME-dependent mechanism [53–57].

Other distinctive features of heme and HO related to one or more so-called hallmarks of cancer have been specifically described in subsequent sections for their particular relevance in breast cancer (BC).

### Predictive and prognostic value of HO-1

It would be highly desirable to exploit HO-1 as a prognostic biomarker. Indeed, its expression has been observed to dictate patients’ prognosis grades in different pathological circumstances, including BC. For instance, HO-1 has emerged as a significant prognostic marker in acute myeloblastic leukemia (AML), particularly in cases involving the Fms-Like Tyrosine Kinase Receptor 3 (FLT3) gene Internal Tandem Duplication (ITD) mutation, which is associated with poor outcomes. The persistent activity of FLT3-ITD in AML cells generates elevated levels of ROS, leading to increased expression of HO-1. Research by Kannan et al. demonstrated that HO-1 expression is significantly higher in FLT3-ITD+ AML cells compared to FLT3-wild type cells, with patients exhibiting elevated HO-1 levels showing reduced survival rates. Additionally, HO-1 overexpression was linked to resistance against the FLT3 inhibitor quizartinib, further highlighting its role in adverse prognosis [58, 59].

Further studies have explored the relationship between HO-1 expression and other molecular factors in AML. Cheng et al. discovered that relapsed AML patients displayed higher HO-1 levels, which were associated with the upregulation of histone deacetylases (HDAC1, HDAC2, and HDAC3) and inversely correlated with the expression of Growth Factor Independent-1 (GFI-1), a transcriptional repressor known to inhibit various malignancies. Notably, low GFI-1 expression was found to upregulate HO-1 through the PI3K/AKT pathway, leading to resistance against Panobinostat, a pan-HDAC inhibitor. This underlines the crucial role of HO-1 in mediating resistance and its potential as a therapeutic target [60].

In a broader analysis, another study confirmed the prognostic value of HO-1 by showing that high-risk AML patients consistently exhibited elevated levels of HO-1 mRNA and protein. Over a three-year follow-up period, these patients had significantly lower overall survival (OS) and relapse-free survival rates, establishing HO-1 overexpression as a predictor of poor clinical outcomes in AML [61].

To further emphasize the prognostic and diagnostic value of HO-1, it’s important to consider its broader implications in various cancers beyond AML. Recent studies highlight that HO-1, along with its upstream regulator Nrf2, plays a critical role in the progression and prognosis of esophageal squamous cell carcinoma (ESCC) as well [62, 63]. In ESCC, overexpression of HO-1 has been strongly associated with poor clinical outcomes, including advanced tumor stages, low tumor differentiation, vascular invasion, and hematogenous metastasis. These findings align with previous observations in AML, furthermore, the co-expression of Nrf2 and HO-1 in ESCC patients has been linked to even worse prognoses, suggesting a synergistic effect in promoting tumor progression and immune evasion. This is particularly evident in high-risk groups where elevated HO-1 levels are predictive of poor progression-free survival (PFS), similar to patterns observed in AML where HO-1 is associated with lower overall survival (OS) and relapse-free survival [64].

Similarly, in non–muscle-invasive bladder cancer (NMIBC), HO-1’s prognostic value has been highlighted in studies examining its expression in relation to tumor grade and recurrence. Increased HO-1 expression, whether at the mRNA or protein level, has been significantly associated with higher tumor grades and a more aggressive disease phenotype. In studies conducted by Yim et al. and Miyake et al., elevated HO-1 levels were linked to poorer relapse-free survival (RFS) and PFS, as well as a higher risk of tumor recurrence [65, 66]. These findings collectively suggest that HO-1 expression could serve as a key marker for identifying high-risk patients in NMIBC, aiding in the prediction of disease progression and informing therapeutic strategies.

In accordance with these findings, HO-1 prognostic value has also been observed for BC. Results reported by Tan et al. highlight the relationship between HO-1 expression and pathological complete response (pCR) outcomes in a cohort of 575 patients with locally advanced invasive BC. Remarkably, they suggest HO-1 as prognostic factor in a neoadjuvant framework, especially in TNBC subgroups, which could help further stratify locally advanced TNBC into subtypes with varying chemosensitivity [67]. Other studies have confirmed these observations suggesting a direct connection with iron-related proteins and HO-1 transcriptional regulators patterns of expression together with iron cycle disruption which provide proof of HO system’s predictive and prognostic value [68, 69]. Nevertheless, the correlation between HO-1 expression and tumor aggressiveness or poor prognosis in BC has not yet been extensively investigated or validated, particularly in comparison to the existing literature on other types of cancer. Although, few studies have been reported in this context, they identify such correlation specifically related to the subcellular localization of the enzyme [70]. Thus, further research is needed to better understand the role of HO-1 in BC progression and to determine whether its expression and localization can serve as reliable prognostic markers or therapeutic targets.

## Breast cancer

BC is a heterogeneous pathology both biologically and clinically with extremely variable outcomes and represents the leading cause of cancer-related death in women. BC comprehends numerous subtypes that differ genetically, pathologically, and clinically. As evidence of this complexity, nowadays it is considered as a group of neoplasms which originate from mammary gland epithelial cells following genetic alterations, displaying different disease courses, responses to treatments, and clinical outcomes [71, 72]. General classification into hormone receptor-positive, human epidermal growth factor receptor-2 (HER2) overexpressing and triple-negative breast cancer (TNBC) is mainly based on histological features. Hormone receptor-positive BC is commonly characterized by increased expression levels of estrogen (ER+) and progesterone (PR+) receptors and/or the proto-oncogene receptor protein tyrosine kinase HER2/neu (HER2+). In particular, HER2 is a member of the human epidermal growth factor receptor (EGFR) family which consists of four members (HER1, HER2, HER3 and HER4) and it is overexpressed in approximately 15–20% of all BCs determining poor outcomes [73]. TNBC, the type lacking expression of ER/PR and HER2/neu proteins, is considered highly metastatic, particularly aggressive, and possesses the worst outcome of all BC subtypes [74].

TNBC represents 15% of all diagnosed BCs and seems to occur with a greater recurrence among ethnic minorities and younger women (<40 years old) showing high histological grade, higher risk of long-term relapses than ER-positive subtypes, alongside frequent spread of metastasis to bone, lung and brain 26557820 [75]. Recently, angiogenic processes have emerged as a promising target for TNBC due to their observed over-activation, which supports tumor growth and primary tumor metastasis spread [76–78]. Although the TNBC subgroup is considered a single entity based on immunohistochemistry (IHC), it was observed a certain heterogeneity that made possible a further classification into other subgroups including basal-like (BL1 and BL2), claudin-low, mesenchymal (MES), luminal androgen receptor (LAR), and immunomodulatory (IM) [79, 80]. Approximately 50–75% of TNBCs show basal-like phenotype (BLBC), however it is not appropriate to identify “TNBC” and “BLBC” as a unique entity, indeed not all BLBCs determined by gene expression profiling (GEP) lack ER, PR, and HER2 and, conversely, not all TNBCs show a basal-like phenotype [81–84].

**Fig. 1:**
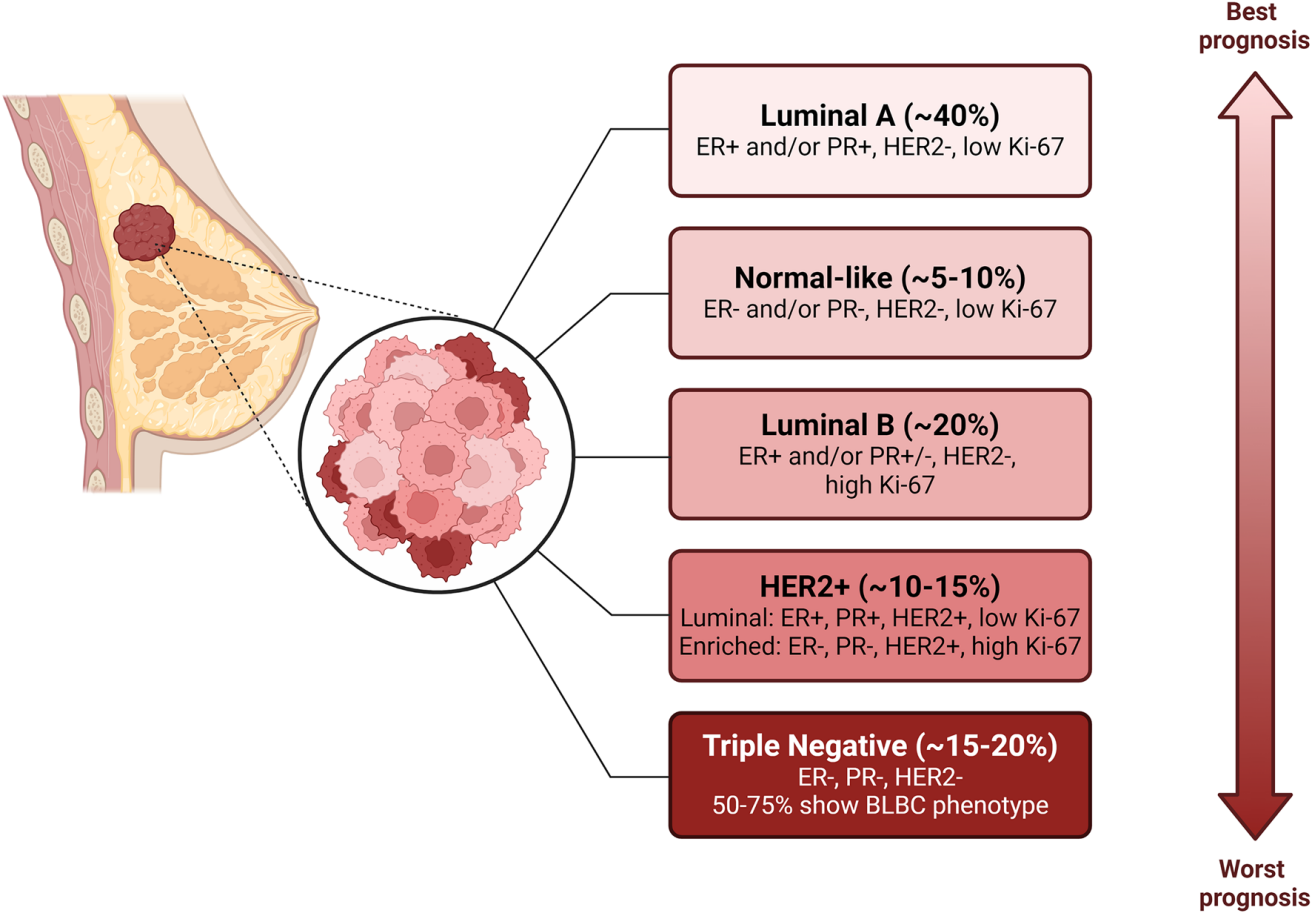
Classification of BC subtypes based on histological features and prognosis entity

The Claudin-low subtype was recently identified and characterized by an active stromal infiltration, different metaplastic histology variants, targetable driver aberrations, low grade of genomic instability and higher expression of epithelial-to-mesenchymal-transition (EMT) genes [85]; however it is still under investigation the correlation between this subtype and a specific clinical outcome or the possibility of using it as a prognostic tool [79]. Based on molecular profiling studies, novel classification of BC has been proposed highlighting 5 subtypes namely luminal A, luminal B, normal-like, HER2-positive and basal-like breast cancers (BLBC) which frequently overlaps with TNBC [86, 87] (Figure 1). Luminal A tumors are the most prevalent subtype (40%) and are mainly characterized by the presence of ER and/or PR and the absence of HER2 and have a low expression of cell proliferation marker Ki67 [88]. Luminal B subtype displays higher grade and worse prognosis compared to Luminal A. They are ER-positive and can be PR negative associated with elevated Ki67 expression, which sustains high proliferation rates resulting in worse prognosis [89]. It represents 10–20% of luminal tumors. Normal-like subtype accounts for about 5%-10% of all breast carcinomas, however it has been poorly characterized. Normal-like cells express signature genes of adipose tissue presenting an intermediate prognosis between luminal and basal-like cancers and don’t respond to neoadjuvant chemotherapy. As ER, PR, and HER2 result absent, these tumors may also be classified as “triple-negative” but lacking Cytokeratin 5 (CK5) and EGFR, they cannot be considered basal-like cancers as TNBC. To date few studies have focused their attention on this subtype, thus its clinical significance remains still undetermined [90]. The HER2-positive subtype comprises 10–15% of all BC and is characterized by absence of ER and PR associated with high HER2 expression. This subtype has a higher proliferation rate compared to the luminal ones, nevertheless the prognosis has improved after HER2-targeted therapies introduction. Within HER2-positive subtype, two subgroups can be distinguished: luminal HER2 (E+, PR+, HER2+, low Ki-67) and HER2-enriched (HER2+, E-, PR-, high Ki-67) [91].

Recent studies showed that BLBC hyper-activate EMT programs and displays several stem cell-like properties, suggesting a pivotal role of EMT transcriptional factors in BLBC progression [92, 93].

The above-mentioned subtypes are also characterized by different prognosis and expected outcomes, indeed luminal A tumors are clinically slow growing and have the most favorable prognosis associated with less incidence of relapse, while HER2-positive and basal-like tumors are associated with low overall survival. Luminal B and normal-like tumors share less severe progression and a better prognosis, additionally these subtypes seem to benefit from hormonal therapy along with chemotherapy. Due to the lack of ER or overexpressed HER2, no targeted therapy is currently available for the BLBC subtype [93].

### Molecular mechanisms associated with BC development

**Fig. 2:**
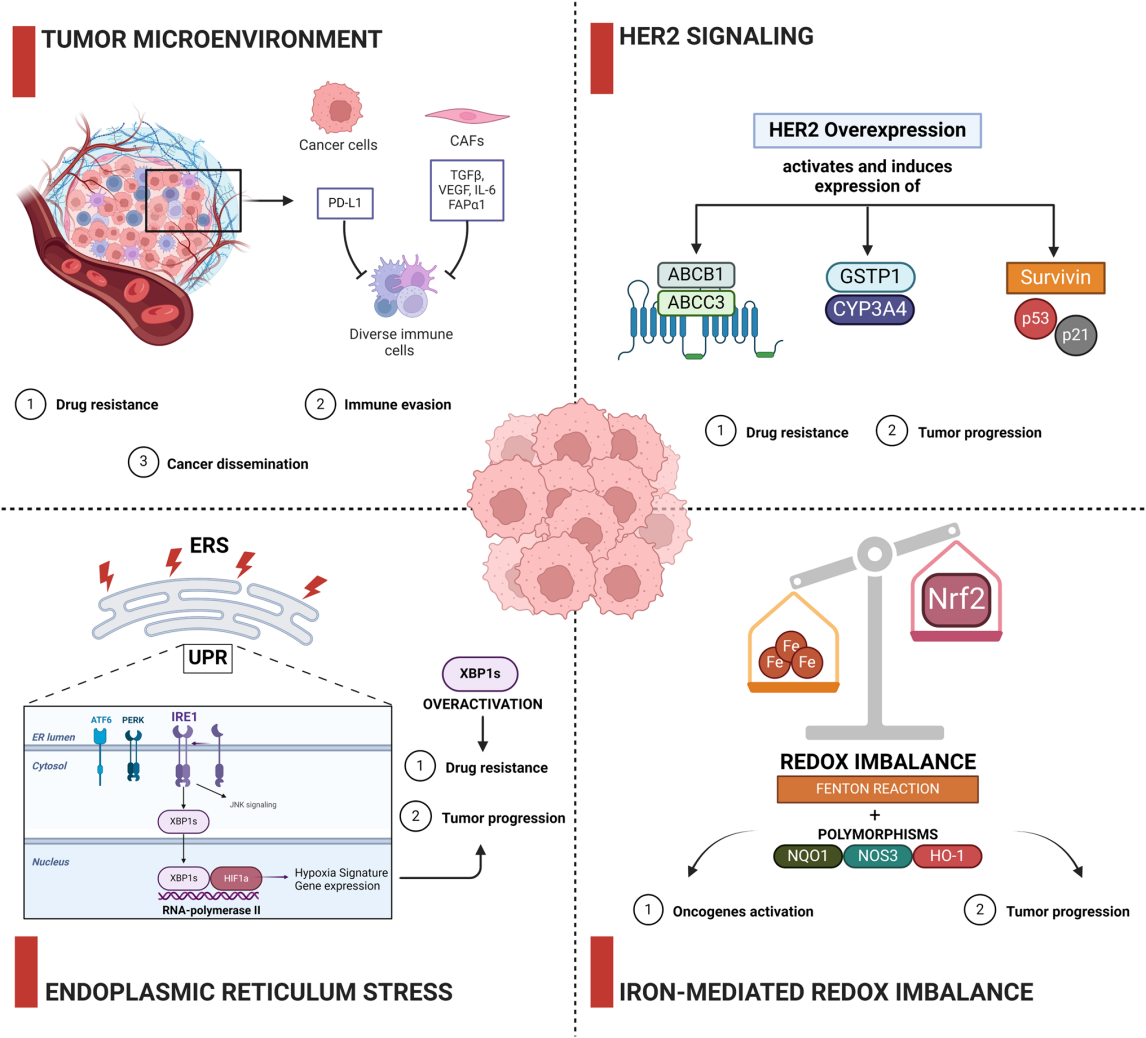
Representation of explored biochemical pathways involved in BC development

BC progression is accompanied by genetic alteration of a multitude of genes which alone or in combination can significantly alter a variety of cellular events. Beside the variations that can be found in different patients’ tumors, even within the same tumor specimen distinct tumor cell populations characterized by independent molecular and phenotypic profiles have been observed, adding a further level of complexity to BC biology. Some of the most relevant pathways involved in BC onset and progression are discussed in the following paragraphs and a schematic representation of them is reported in Figure 2.

#### Tumor microenvironment involvement

The breast tumor mass is composed by a plethora of heterogeneous populations including epithelial cancer cells, but also endothelial cells, cancer-associated fibroblasts (CAFs), and several immune cells forming the so-called tumor microenvironment (TME) [94, 95]. TME is represented by an intricate cellular ensemble within a composite matrix of structural proteins which embodies the extracellular matrix (ECM), where immune cells (including macrophages, mast cells, natural killer cells, dendritic cells), lymphocytes and non-immune cells establish a variety of interconnections with cancer cells. This cellular crosstalk is sustained by the production of specific signals (e.g., growth factors and cytokines), and it determines tumor development [96]. In particular, CAFs, as the most abundant cell-type in TME, secrete substances such as transforming growth factor-β (TGF-β), vascular endothelial growth factor (VEGF), and interleukin-6 (IL-6) which can promote and enhance cancer progression [97]. Substances derived from CAFs play a regulatory role in epithelial-mesenchymal transition and facilitate drug resistance through secretion of interleukin-6 (IL-6) [98]. Moreover, CAFs expressing fibroblast activation protein α1 (FAPα1) are linked to immunosuppression in various cancers, including BC [99, 100]. Following cytokine induction, cancer cells exhibit increased expression of programmed death ligand 1 (PD-L1), the ligand for programmed death 1 (PD-1) protein [101]. Elevated PD-L1 levels in cancer cells contribute to drug resistance, immune evasion and cancer dissemination. The interaction between PD-L1 and PD-1 suppresses T cell function by inducing T cell apoptosis [102]. Hepatocellular carcinoma patients derived CAFs (HCC-CAFs) release IL-6, stimulating PD-L1 expression on neutrophils via the STAT3 pathway [103]. The PD-1/PD-L1 immune checkpoint inhibitors have received approval from the US Food and Drug Administration (FDA) for treating advanced TNBC that expresses PD-L1 [97]. Over the years, specific pathological subgroups have been identified, dictating cancer treatment approaches to follow. Among the multitude of BC subtypes, the Inflammatory breast cancer (IBC) is one of the least common, accounting for approximately 2.5% of BC cases and standing out due to its aggressive and swift tumor development, coupled with a significantly diminished prognosis [104–107]. Moreover, cancer stem cells (CSCs) have increasingly been discussed as potential therapeutic targets in BC and other malignancies [108, 109]. In particular, stem cells have been identified as key mediators of tumor initiation and progression for IBC [110, 111]. In this context, the Musashi (MSI) RNA-binding protein family has been closely associated with cancer stem cells’ activity. As they bind different RNAs to up- or downregulate their translation into proteins, MSI proteins enable modifications of cellular pathways as the Numb/Notch pathway and can alter DNA repair proteins content, therapy resistance, as well as the stemness of cancer cells, highlighting the feasibility of addressing MSI proteins to weaken stem cell traits [107, 112, 113].

#### HER2 signaling

As an oncogene, HER2 is amplified in approximately 25–30% of human breast and ovarian cancers. The overexpression or heightened activation of HER2 triggers aberrant signal transduction pathways that prompt the development of tumors and sustain the growth and survival of malignant cells [114]. Furthermore, HER2 contributes to the drug resistance phenomena in several types of cancer, including BC, sustaining tumorigenesis and cancer aggressiveness. HER2 has also been found to be overexpressed in approximately 13–27% of gastric cancer (GC) cases, correlating with an unfavorable prognosis. Combining monoclonal antibodies as trastuzumab and pembrolizumab has the potential to actively stimulate immune cells, particularly HER2-specific T cells, and significantly alter the immune status of the TME in HER2-positive GC. Thus, the hypothesis of HER2 signaling potential ability to inhibit immune cell infiltration in the TME of those tumor regions rich in HER2 [115]. HER2 is indeed capable of conferring drug resistance by amplifying signals that: (1) prompt the activation of drug-exporting pumps like ATP-binding cassette, sub-family B, member 1 (ABCB1) and ATP Binding Cassette Subfamily C Member 3 (ABCC3); (2) elevate the levels of drug metabolism proteins like glutathione S-transferase P1 (GSTP1) and cytochrome P450 3A4 (CYP3A4); and (3) stimulate the expression of cell survival proteins like survivin, p21, and p53, among others [116]. The heightened signaling by HER2 also leads to increased phosphorylation of estrogen receptor alpha (ERα), resulting in resistance of BC to endocrine therapy. Recent reports indicate that HER2 can also elevate the expression of the breast cancer resistance protein (BCRP) by interacting with EGFR, leading to resistance against aromatase inhibitors [117]. In these cases, the HER2-mediated signal transduction, notably through the PI3K/AKT pathway, is posited as the underlying mechanism behind HER2-mediated drug resistance [118–120].

#### Endoplasmic reticulum stress (ERS)

The endoplasmic reticulum (ER) plays a crucial role in various cellular processes, including the synthesis, folding, and maturation of approximately one-third of the cell’s proteins [121]. However, when the cell is subjected to widespread stress, such as nutrient deprivation, hypoxia, point mutations in secreted proteins, or disruptions in calcium homeostasis, the efficiency of protein folding in the ER can be compromised [122]. This leads to the accumulation of misfolded proteins within the ER, a condition known as ER stress (ERS) [123]. ERS triggers a response known as the unfolded protein response (UPR), which is an adaptive mechanism. The UPR is controlled by three major stress sensors: inositol acquisition enzyme 1α (IRE1α), protein kinase RNA-like ER kinase (PERK), and activating transcription factor 6 (ATF6) [124]. ERS has been implicated in the development and progression of solid tumors, including BC. It has been associated with numerous aspects of BC, such as multidrug resistance, metastasis spread, immunotherapy response, and apoptosis [125, 126], suggesting its critical role in disease’s progression. Recent studies shed light on pivotal role of X-Box Binding Protein 1 (XBP1) gene in oncogenicity, progression, and recurrence of TNBC [127]. Work by Chen et al. indicates XBP1 activation as major driver for BC progression through Hypoxia-inducing factor 1α (HIF1α) transcription control, in particular XBP1 forms a complex with HIF1α leading to the regulation of HIF1α target genes’ expression via the recruitment of RNA polymerase II. Additionally, depletion of XBP1 inhibits tumor growth and relapse in BC cell line models [128, 129]. Overactivation of the XBP1 pathway observed in aggressive TNBC was found to be correlated with HIF1α and hypoxia-driven signatures and that was associated with poor patient survival, highlighting its potential as a therapeutic target [130].

Emerging evidence indicate that the conventional pathological markers (such as ER, PR, HER2, and Ki67) used to predict BC prognosis show certain limitations. To take account of these shortcomings, a prognostic model was developed based on ERS-related scores and clinicopathological factors, including age and TNM (tumour, node, metastasis) stage. Research by Fan et al. demonstrated that patients in the high-ERScore group had significantly worse prognosis, consistently with previous studies on ERS [127]. Additionally, the data indicated that patients with high ERScores exhibited greater resistance to common antitumor agents, and their tumors showed lower levels of immune cell infiltration, suggesting a "cold tumor" state. Prognostic models based on ERS-related scores have shown promise in predicting patient outcomes and understanding treatment resistance and immune responses in BC [131].

#### Iron-mediated redox Imbalance

It is known that a relationship between BC risk and genetic polymorphisms of enzymes involved in the generation and removal of iron-mediated ROS such as Nrf2 (nuclear factor erythroid 2–related factor 2), NQO1 (NAD (P) H Quinone Dehydrogenase 1), NOS3 (nitric oxide synthase 3, also known as endothelial NOS) and HO-1 (heme oxygenase 1) exists and can potentially be used as a pharmacological target for cancer therapies. Indeed, increased oxidative stress promotes Nrf2 nuclear translocation, leading to activated transcription of NQO1, HO-1, NOS3 and other antioxidant response element–driven genes. Enzyme products of these genes act as antioxidant defence reducing iron-generated ROS. Nevertheless, costimulatory relationships exist between HO-1 and NOS3 pathways, fuelling a loop in which nitric oxide induces heme oxygenase (HO) activity, likely through Nrf2 activation, and HO reciprocally stimulates NOS3 activity [132]. Elevated dietary iron intake and substantial iron stores are contributing factors to the generation of ROS in postmenopausal women, indeed high cellular iron content can lead to oxidative tissue damage through the generation of free radicals by Fenton reaction [133–135]. In women, high levels of iron and ferritin have been found only in neoplastic breast tissue and not in normal tissue [136, 137]. Currently, the correlation between excessive dietary iron intake and cancers development, as in the case of lung and colon cancer [138–140], has been supported by epidemiologic studies, but still just few studies have reported significant data for BC risk [141, 142]. Prolonged disruption in the equilibrium of redox processes can potentially influence specific signaling pathways associated with cancer development [143], simultaneously resulting in loss of tumor suppressors and the activation of oncogenes [144]. On the other hand, iron plays a pivotal role in cell proliferation processes, thus it is plausible that iron can also be crucial in the clonal expansion of malignant cells, enhancing tumor progression [145]. Indeed, cancer cells seem to present an iron-deficient phenotype concomitantly with an increase of iron importers’ expression and a decrease of iron exporters [146]. Among women using additional iron supplements, the connections between genetic variations and the risk of tumor insurgence were notably noteworthy only for HO-1. The link between longer HO-1 alleles and BC risk stands as a new discovery, aligning with prior research that has indicated the long HO-1 allele as a predisposing factor for chronic pulmonary emphysema [147], lung adenocarcinoma [148], and oral squamous cell carcinoma [149].

HO-1 induction is assumed to confer cellular protection owing to the reduced presence of pro-oxidant heme, elevated levels of the antioxidant bilirubin [150], and the stimulation of ferritin production, which facilitates swift iron containment [151]. Additionally, HO might contribute to the decrease of intracellular iron levels by promoting the expression of an iron pump that enhances the outward flow of cellular iron [152], and it could mitigate oxidative stress by up-regulating various other antioxidant mechanisms such as superoxide dismutase (SOD), catalase, and NOS3 activity [153, 154]. Nevertheless, HO appears to exhibit dual functions, as evidenced by various studies indicating that its activation doesn’t always yield beneficial outcomes and could potentially contribute to pro-oxidative conditions [155] due to the liberation of ferrous iron and H_2_O_2_ as enzymatic byproducts. This phenomenon could partially elucidate why individuals in the uppermost tier of iron intake, who possess two short HO-1 alleles, demonstrated a marginally heightened, albeit statistically insignificant, susceptibility to BC when compared to LL individuals [132]. This notion is reinforced by findings that homozygous carriers of short genetic repeats are at a higher risk of malignant melanoma compared to carriers of L and M alleles [156].

## Heme metabolism in breast cancer

### Biochemical processes

Heme metabolism refers to the biochemical processes involved in the synthesis, breakdown, and utilization of heme, a crucial molecule in various biological functions. Heme is an iron-containing porphyrin, which is essential for the function of haemoglobin, myoglobin, cytochromes, and other heme-containing proteins, exerting its primary roles in oxygen transport, oxidative metabolism, and xenobiotic detoxification [157]. Heme biosynthesis mainly occurs in the liver and involves a series of enzymatic reactions. The process starts with the condensation of glycine and succinyl-CoA to form 5-aminolevulinic acid (5-ALA), which is further converted into porphobilinogen (PBG) through several enzymatic steps. PBG is then converted into hydroxymethylbilane (HMB), which is further converted into uroporphyrinogen III. The final step involves the conversion of uroporphyrinogen III into heme. This process is tightly regulated and requires various enzymes, cofactors, and iron [158]. As previously mentioned, heme is catabolized by HO, which is found anchored to the ER membrane with the active site facing the cytosol. HOs cleave Fe^3+^-PPIX to form biliverdin, water, carbon monoxide, and iron, which is stored within ferritin. Biliverdin is subsequently converted to bilirubin by biliverdin reductase using NADPH as a cofactor [157]. Heme is a vital component of various proteins and enzymes in the body. Hemoglobin, for example, is essential for oxygen transport in red blood cells, while cytochromes play crucial roles in electron transport and oxidative phosphorylation in mitochondria. Myoglobin, found in muscle cells, facilitates oxygen storage and release. Additionally, heme-containing enzymes, such as catalase and peroxidase, are involved in antioxidant defence mechanisms.

### Implications in BC progression

**Fig. 3:**
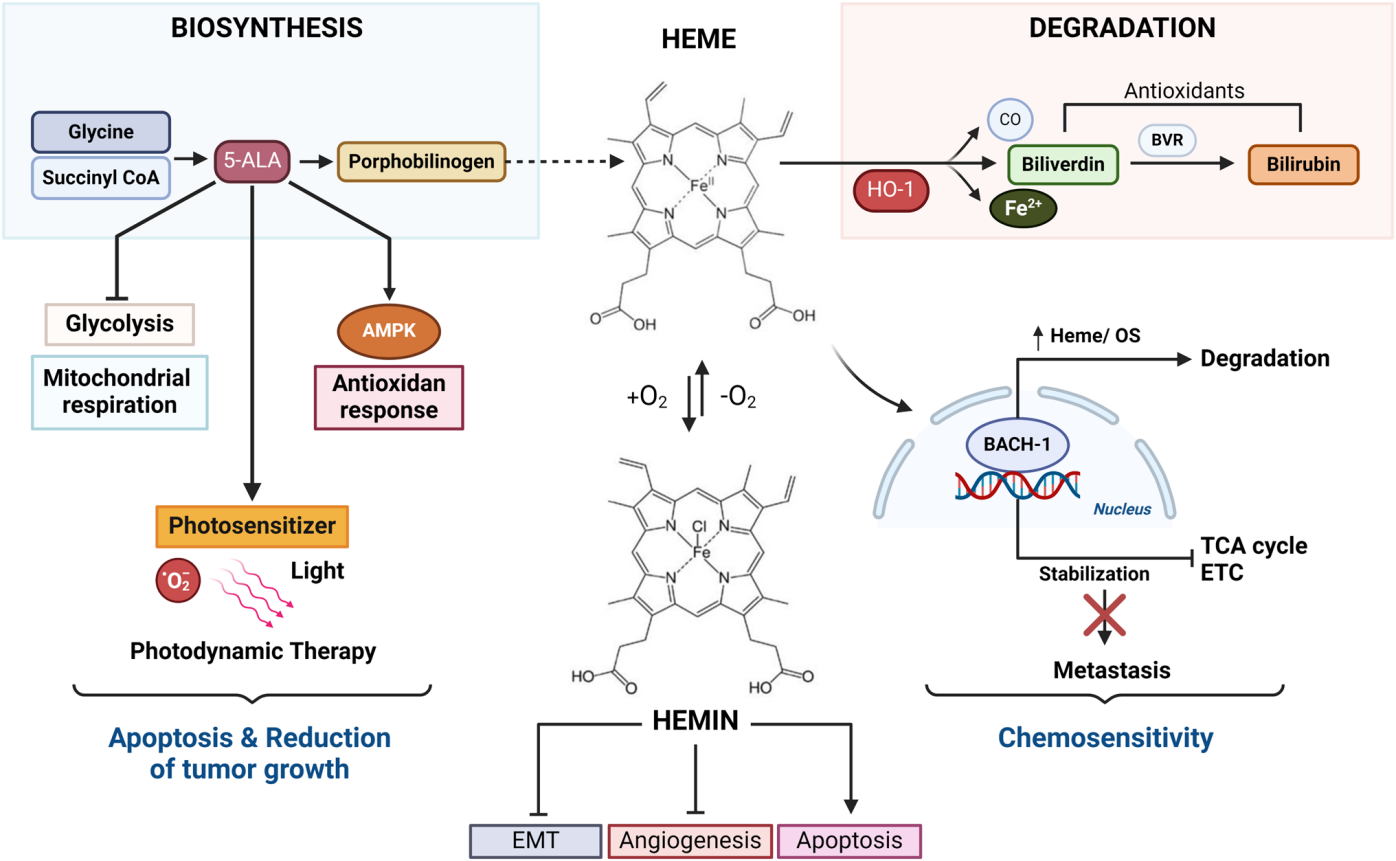
Schematic representation of heme metabolism implications in key metabolic processes involved in cancer

Regulation of heme metabolism in BC is crucial as alterations in heme synthesis can impact pathways related to energy generation, anabolism, and oxidative stress. Additionally, the activation of heme synthesis has been shown to regulate glycolysis, electron transport chain (ETC), oxidative phosphorylation (OXPHOS), and the tricarboxylic acid (TCA) cycle in BC cells, highlighting the intricate interplay between heme metabolism and key metabolic pathways involved in cancer cell growth and survival (Figure 3) [159].

#### Hemin

Research indicates that hemin, the oxidized form of heme, exhibits antitumor activity in BC by modulating protein expression and affecting various biological functions within cancer cells. Hemin treatment has been shown to significantly alter the expression of proteins related to heme, iron, adhesion, cytoskeleton, signal transduction, and lipid metabolism in BC cells, highlighting its multifaceted impact on cancer physiology [160]. Moreover, hemin has demonstrated the ability to regulate pathways involved in cell survival and proliferation, including the PI3K/Akt signaling pathway and the NF-kB pathway, which may impact processes like EMT, angiogenesis, and apoptosis in BC, suggesting its potential as a therapeutic agent towards tumor progression [161]. In patients bearing TNBC, a correlation between cancer progression and inducible nitric oxide synthase (iNOS) overexpression was observed [162–164]. Indeed, excessive NO production affects tumor size and blood supply. In this context, hemin was proposed as a potential NO-scavenging agent, able to inhibit TNBC cells migration and tumor aggressiveness linked to EMT processes [165]. These combined effects were also observed to be strictly correlated to HO-1 increased expression and activity, suggesting its additional modulatory role in EMT within different BC cell lines [166, 167].

#### 5-aminolevulinic acid (ALA)

Promoting heme production through ALA supplementation results in the decrease of glycolysis, mitochondrial respiration, and cell growth in ovarian and BC cells. SKOV3 cells exhibited reduced activity in glycolytic and aerobic metabolism compared to MDA-MB-231 cells, and their proliferation was more significantly inhibited by ALA treatment at lower concentrations. The underlying mechanisms involve destabilization of Bach1, activation of AMPK, and stimulation of antioxidant response. This implies that cancer cells relying more on aerobic metabolism are less responsive to ALA treatment. BC patients were found to have abnormally elevated carboxyhaemoglobin levels (2.5 ± 1.3%), indicating HO-1 upregulation in BC cells [159, 168].The use of 5-ALA in cancer therapy, particularly for BC treatment, has shown promise as a photosensitizer for photodynamic therapy (PDT). PDT uses light to activate a photosensitizing agent like 5-ALA in the presence of oxygen, leading to the production of reactive oxygen species (ROS) that can potentially induce apoptosis in cancer cells. Recent research suggests that employing 5-ALA-PDT may enhance the sensitivity of BC cells to tamoxifen, a commonly used hormone therapy medication for this type of cancer [169, 170]. Additionally, inhibiting the ATP Binding Cassette Subfamily G Member 2 (ABCG2) transporter may reverse resistance of TNBC cells to ALA-PDT. Membrane solute carrier transporters like SLC46A1 and SLCO1A2 are suggested to play a role in BC prognosis by transporting anticancer drugs and physiological substrates. Furthermore, free heme not incorporated into hemoproteins catalyzes free radical production through Fenton chemistry, while elevated synthesis or accumulation of heme intermediates may trigger increased ROS synthesis [171].

#### Bach-1

BTB domain and CNC homolog 1 (Bach-1) is a transcriptional regulator of several glycolytic genes and it has been found to possess the ability of repressing transcription of TCA cycle and ETC related genes in BC [172–174]. Additionally, it is involved in cell migration processes and metastasis dissemination [175, 176]. Heme intracellular levels can affect Bach-1 stability and stimulate its ubiquitination, under balanced redox conditions Bach-1 acts as a negative regulator of antioxidant genes transcription, including HO-1, by binding antioxidant response elements (AREs) in their promoter regions [177]. The opposite situation occurs under oxidative stress conditions, indeed free heme released from hemoproteins contribute to the increase of ROS levels and directly affect Bach-1 activity by inducing its degradation. High levels of Bach-1, together with its increased stabilization, have been reported in lung cancer metastasis and BC cells. In particular, Bach-1 stabilization in TNBC leads to lower TCA cycle activity and reduction of ETC genes levels, thus undermining its stabilization may sensitize TNBC cells to ETC inhibitors such as metformin [159, 174]. In TNBC patients, upregulated Bach-1 was correlated with short disease-free survival and tumor stage [68, 178], moreover it was also reported a direct correlation with overexpression of long non-coding RNA small nucleolar RNA host gene 5 (SNHG5) which is responsible for enhanced glycolysis and growth of cancerous cells [179]. Additionally, Bach-1 was observed to enhance matrix metalloproteinases (MMPs) and CXC-chemokine receptor 4 (CXCR4) mRNA serving as transcriptional regulator by interacting directly with promoter regions of target genes and promoting metastasis in BC cells [180].

## Heme oxygenase roles in breast cancer

Understanding the intricate interplay between the HO system and BC could provide valuable insights into potential therapeutic interventions. Genetic variations within the HO-1 promoter have been linked to an elevated risk of cancer progression and heightened rates of treatment ineffectiveness. Furthermore, data obtained from processed cancer biopsies point out the potential association between HO-1 expression, pathological characteristics, and clinical outcomes [68]. As HO-1 expression has been observed to be modulated also at a post-transcriptional level, microRNAs (miRNAs) seem to play a key role [70]. Indeed, miRNAs can affect HO-2 expression both directly or indirectly, through Nrf2 modulation [181]. Recent studies have demonstrated miRNAs involvement in the regulation of HO-1 in cancer cells. For instance, miR-155 has been found to promote resistance to arsenic trioxide in lung cancer through the activation of Nrf2/HO-1 signaling pathway [182]. Similarly, miR200a has been identified as a regulator of HO-1 expression via Nrf2 activation by targeting Keap1 mRNA in BC [183]. Interestingly, HO-1 protein has also been found in extracellular vesicles collected from the culture medium of several types of cancer cells, including BC [184]. Recently, there has been a growing body of evidence suggesting that cancer is not exclusively driven by genetic factors, but it involves metabolic dysregulation too. Oncogenic signaling pathways are now recognized to play a role in both catabolism and anabolism, crucial for fuelling the rapid growth of tumors [185]. This perspective has established metabolic reprogramming as a signature feature of cancer. In particular, TNBC cells exhibit distinct metabolic characteristics such as elevated glycolysis rate and reduced mitochondrial respiration which confer particular sensitivity to glycolytic inhibition, proposing such metabolic intervention as effective therapeutic strategy for this subtype of BC cells. However, HER2-positive tumors demonstrate increased lipid metabolism and glutamine metabolic activity compared to other BC subtypes [186, 187]. Additionally, studies by Oshi et al. in two large cohorts of BC patients with high glycolysis scores showed a poor prognosis in TNBC subtype, but not in ER-positive/HER2-negative BC, potentially suggesting that counterbalance of other distinct hallmark of BC subtypes signaling may affect patient prognosis [188]. Research indicates that HO-1 expression can impact the efficacy of anticancer therapies, with its overexpression being associated with poor prognosis in BC patients undergoing chemotherapy [189, 190]. Inhibition of HO-1 has been shown to enhance the effectiveness of anticancer therapy and inhibit tumor growth due to its modulating activity towards immunosuppressive TME [191]. Specifically, breast tumor cell debris-induced HO-1 expression in tumor-associated macrophages (TAMs) has been linked to reduced efficacy of paclitaxel, a common anticancer drug used in BC treatment. Conventional chemotherapy is capable of generating significant amounts of tumor cell debris which can accelerate the immunosuppressive M2-like macrophage polarization in TME. Thus, TAMs’ HO-1 inhibition in the context of BC therapy could potentially lead to improved treatment outcomes by enhancing the effectiveness of chemotherapy and inhibiting tumor progression through HO-1-targeted reprogramming toward the M1 phenotype [192]. Work by Muliaditan and colleagues shed light on the potential role of HO-1 as an immune checkpoint proposing tin-mesoporphyrin (SnMP) as a novel immune checkpoint inhibitor to be used in combination with chemotherapy allowing immunological control of tumor growth in different preclinical models of BC. Indeed, SnMP counteracts chemotherapy-elicited CD8+ T cells immunosuppressive effects through myeloid HO-1 activity inhibition. Evidence reported in this study elicits HO-1 potential as an innate checkpoint compared with PD-1 and CTLA-4, as it does not require receptor-ligand interactions. Moreover, *in vivo* data highlight the therapeutic efficacy of SnMP which compares favorably with anti-PD-1 blockade within chemotherapy regimens [193]. *In vivo* studies support the potential use of combinatory treatment of anti-PD-1 agents and HO-1 inducers as it was shown that especially in HO-1-overexpressing BC they result effective. Additionally, in the attempt to gain mechanistic insights on patients’ different responses to immunotherapy, the correlation between higher levels of total cholesterol, low-density lipoprotein (LDL), and oxidized low-density lipoprotein (ox-LDL) with reduced CD8+ T cell effector function was observed, and deeper investigation led to the acknowledgment of HO-1 involvement. Indeed, ox-LDL induced HO-1 expression reducing tumor response to immunological treatment as the cell activates cytoprotective stress responses [194].

### HO-1 cellular localization

Since its discovery in 1968, HO has been identified as an ER-associated protein whose activity was observed to be highly detected in microsomal fractions. Through the years, HO-1 was also detected in other subcellular compartments such as mitochondria, plasma membrane, vacuole and nucleus. In most of the cases HO-1 maintains its enzymatic activity, except for the case of nuclear translocation, indeed HO-1 loses its catalytic function and gains a transcriptional role [70, 195]. HO-1 translocation into the nucleus is known to occur through signal peptide peptidase (SPP)-mediated cleavage, resulting in a C-terminal truncated form of HO-1 which losts its catalytic ability acquiring a transcriptional activity due to nuclear interaction and stabilization of Nrf-2 [196, 197]. Several studies have already confirmed HO-1 relevance in many types of tumors, including BC, however HO-1 nuclear translocation leads to potentially opposite changes in cellular behavior suggesting a context dependency which involves tumor grade, tissue specificity, HO-1 threshold levels together with its sub-localization [198–200]. Despite no evident correlation was found between HO-1 nuclear localization and survival, it was reported an association with higher histological grade suggesting its potential role in more aggressive tumors. This hypothesis was also accompanied with the idea of HO-1 acting primarily as the protein itself, regardless of its enzymatic by-products’ activity. Interestingly, it was observed the correlation between poor overall survival of BC patients and increased expression of cathepsin B, calpain-2 and SPP, which represent the proteases responsible for C-terminal cleavage of HO-1. HO-1 clinical significance has been revealed in BC tissues as its increased protein levels were correlated with better patients’ prognosis, reduced tumor size and longer overall survival rate. However, HO-1 nuclear localization was observed to be associated with higher tumor grades suggesting peculiar activity depending on its cellular sub-localization. *In vivo* evidence reported a reduction in tumor burden of different BC animal models and metastatic dissemination following both HO-1 pharmacological activation and genetic induction. Additionally, the same results were obtained *in vitro* using BC cell cultures, indeed HO-1 induction led to reduced cell viability due to cell cycle arrest and apoptosis activation, together with affecting EMT-related pathways [200, 201]. While HO-1 is commonly seen as a protective molecule that helps cells survive stress, it can also act as a mediator of cell death, probably through a process called ferroptosis. This dual nature implies that HO-1 can either promote cancer progression or inhibit it, depending on the context and conditions within the tumor microenvironment. The complex regulatory mechanisms that control HO-1 expression and activity pose challenges in developing effective therapies that specifically inhibit HO-1 without causing unintended side effects or disrupting normal cellular functions. Understanding the intricate balance of HO-1 functions and its interactions within the tumor microenvironment is crucial for overcoming these challenges and developing successful targeted therapies for BC.

## Advancements in Breast Cancer Therapeutic Strategies

### Conventional therapies

Given the elevated occurrence of BC beyond the age of 30, it’s essential to meticulously weigh treatment options during the reproductive years (between 15 and 49) as significant life milestones like pregnancy and childbirth often coincide within this timeframe [202]. The conventional treatment for hormone-responsive BC is represented by endocrine therapy through administration of aromatase inhibitors, selective estrogen receptor modulators (SERMs) and selective estrogen receptor down regulators (SERDs). HER2-positive BC patients undergo chemotherapy associated with monoclonal antibody (mAb) trastuzumab as first-line treatment. In recent years, several HER2-targeted protocols have been established including the use of other mAbs as pertuzumab and small molecules as lapatinib, neratinib, and sapatinib, all classified as receptor tyrosine kinase inhibitors (RTKi) [203, 204]. In the case of TNBC, fully successful or specific regimens have not yet been established, thus the current protocol also involves a combination of chemotherapeutic drugs and RTKi which does not always lead to higher overall survival as long-term efficacy remains poor [78, 205]. Over the past decade, the U.S. Food and Drug Administration (FDA) has approved several novel targeted therapies, such as cyclin-dependent kinase (CDK) 4/6 and poly (ADP-ribose) polymerase (PARP) inhibitors, offering valid treatment alternatives for individuals diagnosed with BC. A comprehensive review on this topic was provided by Arora et al. reporting all medications FDA approved for BC and how it was possible to reach approval in order to satisfy unmet clinical needs [206]. The first therapies, specifically approved for TNBC, involve Atezolizumab [207, 208], Pembrolizumab [209], Sacituzumab govitecan-hziy [210], respectively, two anti-PD-1 monoclonal antibodies and an antibody-drug conjugate (ADC) directed against human trophoblastic cell surface antigen 2 (Trop-2). ADCs have been rapidly arising as promising treatments for various solid tumors, however, part of patients under ADCs regimens can develop resistance to these chemotherapeutic agents leading to a progression of the malignancy. In particular, evidence collected in recent years seems to suggest among the main causes of resistance insurgence the following factors: dysfunctional intracellular signaling, rapid loss of antibody mediated activity and enhanced expression of drug efflux transporters. Thus, it is reasonable to administer a combination of ADCs and other targeted-therapeutics showing synergistic mechanisms in order to improve therapeutic effectiveness and increase patients survival rates, or at least delay chemoresistance onset [211–213]. Among the combination tested in different clinical trials, Trastuzumab drug conjugates (T-DCs) and immune checkpoint inhibitors (ICIs) together with association of T-DCs and poly (ADP-ribose) polymerase (PARP) inhibitors were reported to be effective as it was observed a significant impairment in DNA repair mechanism leading to higher protocols’ success [214, 215]. Additional encouraging evidence was reported in the case of lapatinib and ado-trastuzumab emtansine (T-DM1) combination in both early and advanced stage of BC [216].

### HO-1 modulating therapies

Scientific evidence has shown that HO-1 induction in response to chemotherapy can reduce its efficacy, leading to cell survival and resistance. Among the anticancer agents able to induce HO-1 through high ROS production are etoposide, doxorubicin, and pharmorubicin [217–219]. Notably, HO-1 increase not only leads to reduced chemotherapeutics efficacy, but it is also reported to be involved in radio- and photodynamic therapies resistance, for instance in non-small cell lung cancer carcinoma [220, 221]. Indeed, HO-1 therapeutic significance has been widely highlighted for several types of cancer [60, 222–225]. Nonetheless, there has been limited analysis of its impact on BC specifically. Therefore, further investigation and expansion of this research area are warranted. In this context, pharmacological and gene-editing tools for the modulation of HO-1 have been proposed in order to potentially translate their use in clinical studies [226]. However, it must be kept in mind that HO-1 modulation cannot be effective as a single therapy, but most likely as a coadjuvant therapy to potentially increase tumoral cells sensitivity to conventional treatments. To date, two generations of HO-1 inhibitors have been developed, respectively metalloporphyrins and imidazole-based compounds, representing the main pharmacological tools. Briefly, metalloporphyrins (MPs) as zinc protoporphyrin (ZnPPIX), tin protoporphyrin (SnPPIX), or chromium protoporphyrin (CrPPIX) have been widely characterized and in the years some MPs limitations, such as the poor water solubility, have been overcome thanks to conjugation with polyethylene-glycol or amphiphilic styrene-maleic acid copolymer (PEG-ZnPPIX and SMA-ZnPPIX, respectively) [227, 228]. Nevertheless, major drawbacks such as photoreactivity and low selectivity for HO-1 isoform remain unsolved [229–231].

The second generation of HO-1 inhibitors, with the first imidazole-based compound being Azalanstat (or QC-1) [232], has been growing in the last decade as several new compounds have been synthesized starting from compounds QC-13 and QC-308 [233, 234]. The proposed mechanism of action of imidazole-dioxolane derivatives relies on their characteristics of being non-competitive agents as they bind the distal side of heme impairing the enzyme catalytic activity [235]. Potent antitumor effect of imidazole-based HO-1 inhibitors was confirmed *in vitro* in several types of cancer, including BC [27, 236–238]. Recent advances in studies of imidazole-based HO-1 inhibitors have also been obtained by developing novel mutual prodrugs proving their effectiveness in synergistically enhance some standard anticancer agents’ cytotoxicity, increasing tumorous cells sensitivity while reducing harmful effects on healthy cells [239–241]. However, the limitation of this class of compounds consists of lacking *in vivo* investigation and information on pharmacokinetics, biodistribution, and safety profile. In order to overcome this liability, novel drug discovery studies have led to the development of a next generation HO-1 inhibitor called KCL-HO-1i which is orally bioavailable in murine models with an observed serum half-life of 3hours. Bahri et al. for the first time demonstrated that HO-1 inhibition via KCL-HO-1i in a particular subpopulation of TAMs has the ability to switch from an immunological ‘cold’ to ‘hot’ TME which should improve the response to chemotherapeutic drugs, highlighting the immunotherapeutic potential of this promising new compound [242]. For what concerns HO-1 induction, this strategy has been recently investigated in the context of a novel type of cell death described in 2012 by Dixon et. al defined as Ferroptosis [243]. The process of ferroptosis is primarily distinguished by a substantial iron-dependent generation of ROS, leading to the subsequent peroxidation of phospholipids (PLs) containing polyunsaturated fatty acids (PUFAs) through the Fenton reaction. Indeed, ferrous iron released by augmented catalytic activity of HO-1 can enhance ferroptosis onset, thus its pharmacological induction has been proposed as an alternative strategy for cancer treatment [244]. Even in this case, HO-1 targeted therapy cannot provide a unique solution or replace conventional chemotherapeutics but seems to be an intriguing and convenient combination strategy. Indeed, recent studies proved the efficacy of siramesine and lapatinib co-treatment in different breast carcinoma cell lines (MCF-7, MDA-MB-231, ZR-75, and SKBr3) through activation of ferroptotic pathways [245]. Furthermore, it was also reported that ferroptosis induction was able to restore gefitinib sensitivity in gefitinib-resistant TNBC cells [246]. For this purpose, several studies have been focusing on phytochemicals ability to induce ferroptosis and enhance chemotherapy efficacy [33, 247], indeed many natural compounds are able to induce both HO-1 expression and enzymatic activity serving as promising coadjuvant for BC treatment [248–251].

### Innovative therapies

While conventional therapies such as surgery, chemotherapy, and radiation therapy have contributed to improved outcomes for many patients, typical challenges such as treatment resistance, disease recurrence, and adverse side effects persist. In response, researchers and clinicians continue to explore innovative approaches to BC treatment, seeking to enhance efficacy, minimize toxicity, and address unmet clinical needs. In the following paragraphs, were presented some innovative techniques and strategies resulting from advances in biotechnological and medical knowledge that we have deemed most compelling to highlight the innovative direction that has been pursued in recent years in the field of oncology.

#### CART-T cell therapy

CAR-T cell therapy involves using chimeric antigen receptor (CAR) T cells to target specific antigens present on BC cells. This innovative immunotherapy approach harnesses the patient’s own immune cells to combat cancer. Various tumor-associated antigens have been identified as potential targets for CAR-T cell therapy in BC, including HER2, EGFR, HGFR/cMET, ROR1, AXL, MUC1, mesothelin (MSLN), and others [252, 253]. Clinical trials are ongoing to evaluate the efficacy and safety of CAR-T cell therapy in BC, aiming to improve treatment outcomes and overcome challenges such as insufficient trafficking, immunosuppressive environments, and toxicities associated with CAR-T cell therapy. Further research and clinical trials are needed to advance CAR-T cell therapy for patients and enhance its therapeutic efficacy [253–255]. The therapy has shown promising results in hematologic malignancies but faces challenges in solid tumors like BC. Toxicities and intratumor antigen heterogeneity represent important issues to be addressed to improve clinical efficacy of CAR-T cells in solid tumors, in particular, side effects as massive cytokines release, multiorgan toxicity and insurgence of encephalopathy syndrome have been linked to CAR-T cells therapy, limiting the potential benefits of the protocol [256, 257]. Additionally, complexes and differentiated cancers, as BC, may expose different CAR-redirected target antigens on their surface or not express the target antigen at all, leading to a failure in detecting malignant cells with consequent loss of efficacy [258, 259]. Interesting findings have been obtained in preclinical models of BC using CAR-T cell-derived exosomes [260]. In particular, these nano-sized vesicles are capable of delivering several active effectors as proteins, granzymes and perforins together with mRNA and miRNA, acting as crucial messengers [261]. Indeed, it has been reported that surface membrane antigens deriving from parental cells are also carried by isolated CAR-T cell exosomes. CAR-T cell-derived exosomes elicited indeed a significant immune response in BC cells accompanied by apparent lower cytotoxicity compared to CAR-T cells due to reduced cytokines release. In this context, modulating heme metabolism and HO-1 activity has been proposed as a potential strategy to improve the tumor microenvironment and enhance the efficacy of cancer immunotherapies as CAR-T cell therapy. Indeed, results reported by Gupta et al. show heme ability of increasing effector T and CAR T cell function while decreasing T cell exhaustion, leading to cytotoxicity of heme-supplemented CAR T cells [262].

#### Photodynamic therapy (PDT) & photothermal therapy (PTT)

Photodynamic therapy (PDT) is a well-established minimally invasive treatment for various diseases, including cancer. Clinical studies have demonstrated the potential curative effects of PDT, especially in the case of early-stage tumors. Additionally, it has been shown to extend survival rates among patients with inoperable cancers and notably enhance their overall quality of life [263].

PDT involves the administration of a photosensitizer followed by irradiation with specific wavelengths of light, leading to the production of cytotoxic singlet oxygen molecules [264]. Hemin, also known as HiPorfin in clinical applications, plays a crucial role in PDT. It can convert oxygen molecules into cytotoxic singlet oxygen under specific laser irradiation, causing DNA damage and inducing apoptosis in cancer cells [265]. Furthermore, hemin serves as a peroxidase, improving the hypoxic conditions in tumors and enhancing PDT efficacy. In a recent study, a dual-controlled novel smart nanoplatform loaded with a Pt^IV^ derivative of cisplatin (CDDP) and triptolide (TC) as an axial ligand was developed for enhanced TNBC treatment. This nanoplatform, known as HTC@ZIF8, releases hemin and the Pt^IV^-triptolide derivative (TC) under acidic tumor microenvironments [266]. The released hemin generates ROS, inducing irreparable DNA damage and ferroptosis while the CDDP prodrug (TC) depletes glutathione (GSH) and inhibits the activation of glutathione peroxidase 4 (GPX4), promoting ferroptosis. Additionally, the released triptolide derivative suppresses GSH levels by regulating Nrf2, further enhancing membrane lipid peroxidation. Recent studies have highlighted the relevance of combining PDT and photothermal therapy (PTT) to eradicate cancer BC. Near-infrared (NIR) organic small molecule indocyanine green (ICG) was used to create injectable agarose *in situ* forming NIR-responsive hydrogels (CIH) incorporating Cu-Hemin and then the nanoplatform was tested both *in vitro* and *in vivo*. The ICG hydrogel stimulated with an 808 nm laser was able to convert light energy into thermal energy, resulting in the heating and softening of the hydrogel matrix promoting subsequent release of Cu-Hemin, showing surprisingly a certain grade of safety [267]. Another potential use of hemin for PDT and synergistic PTT was provided by Chen et al., whose work highlighted the efficacy of biocompatible nanosystems integrating hemin into black phosphorus nanosheets for BC therapy. In particular, hemin characteristic feature of enhancing PDT and reducing GSH leads to a reduced hypoxic condition of the TME promoting higher efficacy of the treatment. Moreover, combination with PTT showed higher antitumor effect through inflammatory factors activation and adequate immune response [268].

#### DNA origami

The advent of DNA origami technology, where long viral ‘scaffold DNA strands’ are folded with chemically synthesized ‘staple DNA strands’ into precise 2D and 3D shapes, has transformed the landscape of DNA nanotechnology [269]. In the construction of DNA nanosystems, the precision of Watson–Crick base pairing serves to manipulate materials at the nanoscale. Employing this approach for drug delivery, holds significant promise for maximizing therapeutic efficacy while minimizing adverse effects on healthy tissues, facilitating the creation of intricate assemblies that combine drugs, targeting ligands, and other functionalities within a single nanostructure. Anticancer drugs like Doxorubicin (DOX), which intercalate with DNA, exhibit selective binding to DNA nanostructures. This property makes DNA origami structures excellent nanocarriers for targeted and precise delivery of anticancer medications. Some examples of this sophisticated and advanced technology have been applied in preclinical studies for the development of innovative BC treatments [270, 271]. In 2012 Zhao and colleagues designed DNA nanostructures for optimal delivery of the anthracycline DOX to human BC cells, proving them to be efficient delivery systems which enhanced Dox internalization to induce apoptosis in BC cells at lower concentrations than those required for free DOX [272]. An upgrade of this type of nanocarriers was obtained by folate-functionalized DNA origami structure design, which demonstrates enhanced efficacy in targeting and eliminating folate receptor alpha (FOLR1) overexpressing TNBC cells compared to non-targeted origami [273]. DNA nanostructures’ versatility makes them extremely appealing for biomedical application and their functionalization with targeting agents like antibodies, affibodies, and aptamers allow to improve specificity and efficacy. By immobilizing antigens on DNA origami surfaces, antibody-antigen interactions can be studied, enabling conformational changes that facilitate cargo display, such as drug molecules. As emerged, targeting strategies using DNA-based tools show promise in cancer treatment, exhibiting enhanced inhibition of malignant cell growth and efficient drug delivery. Nevertheless, their structural stability may be compromised in challenging environments as nuclease-rich physiological conditions, hence the arising of studies proposing various coating strategies. An innovative approach was developed using a facile, modular, stimuli-responsive, two-component coating system for DNA origami nanostructures consisting of bovine serum albumin (BSA) and anti-human epidermal growth factor 2 single-chain antibody fragment (anti-HER2) conjugated to a second generation Newkome-type dendron (G2), in order to obtain both targeting and camouflaging features [274, 275].

## Conclusions

The literature herein collected highlighted the underlying significance of heme metabolism and HO-1 in BC research as demonstrated by increasing evidence of their involvement both in disease onset and progression, identifying them as novel potential targets for the treatment of this malignancy. Additionally, a brief look on how innovative therapeutics are going to shape the future concept of precision medicine has been reported, including the potential of new tools to intercept heme metabolic pathway and in particular modulate HO-1. Interestingly, recent work by Alsharabasy and colleagues investigates hemin’s potential as anticancer agent in BC cells. They showed hemin and its derivatives, hemin-tyrosine (H-Tyros), and hemin-styrene (H-Styr), efficacy in promoting HO-1 expression with consequent pro-oxidative effects and cell migration reduction. Molecular docking simulations reveal how hemin and related compounds interact with HO-1, offering insights into oxidative stress regulation and suggesting that controlled delivery of hemin or its analogs has the potential of leading the way to the development of innovative targeted BC therapies [276].

While several reviews have covered iron or heme topics, few have addressed the potential role of heme oxygenase and heme-related pathways in BC. Therefore, a significant question remains unanswered in the future of BC treatment. Thus, the intent of this work is to emphasize on recent findings which have prompted research in the field exploring novel frontiers of therapeutical interventions.

## Data Availability

No datasets were generated or analysed during the current study.
